# Integrin α4 and α9 in trabecular meshwork and Schlemm’s canal function: emerging regulators of intraocular pressure homeostasis

**DOI:** 10.3389/fcell.2026.1862957

**Published:** 2026-07-23

**Authors:** Tania Sharmin, Colleen M. McDowell

**Affiliations:** Department of Ophthalmology and Visual Sciences, School of Medicine and Public Health, University of Wisconsin-Madison, Madison, WI, United States

**Keywords:** extracellular matrix, fibronectin, fibrosis, glaucoma, integrins, IOP, Schlemm’s canal, trabecular meshwork

## Abstract

Integrins are hetero-dimeric transmembrane receptors that facilitate bidirectional signaling between the extracellular matrix and the intracellular environment. Among these, the α4 and α9 subunits form a distinct subfamily when paired with the β1 integrin subunit. α4β1 and α9β1 integrins play specialized roles in cell migration, adhesion, and mechanotransduction. In the ocular anterior segment, these integrins are recognized for their contributions to the physiological function of the trabecular meshwork (TM) and Schlemm’s canal (SC). This review examines the structural and functional characteristics of α4β1 and α9β1, their involvement in aqueous humor (AH) outflow and intraocular pressure (IOP) regulation, and their specific interactions with the fibronectin extra domain A (FN+EDA). Furthermore, we explore the crosstalk between these integrins and the toll-like receptor 4 (TLR4) and transforming growth factor-beta (TGFβ) signaling pathways, highlighting their potential role in driving a fibrotic-like response observed in the glaucomatous TM.

## 1 Introduction

The regulation of intraocular pressure (IOP) is fundamental to ocular health, primarily managed by the balance between aqueous humor (AH) production and its drainage through the conventional outflow pathway (van Zyl et al., 2020). This pathway comprises the trabecular meshwork (TM), and the endothelial lining of Schlemm’s canal (SC) (van Zyl et al., 2020; Aspelund et al., 2014; Kizhatil et al., 2014). Dysfunction within these tissues, often characterized by excessive extracellular matrix (ECM) deposition and increased stiffness, leads to elevated outflow resistance and ocular hypertension, the primary risk factor for the development of primary open-angle glaucoma (POAG) (Last et al., 2011; Wang et al., 2018; Lü et al., 1981).

Integrins are heterodimeric transmembrane receptors consisting of one α-subunit and one β-subunit that serve as the primary mechanical link between the ECM and the cytoskeleton (Tamkun et al., 1986; Argraves et al., 1987). In humans, 18 α and 8 β-subunits combine to make up 24 distinct integrin heterodimers which facilitate cell–cell and cell–ECM interactions and signaling, thereby playing important roles in several cellular processes, most notably in adhesion, migration, survival, proliferation, and differentiation (Hynes, 2002).

The function and role of several integrins have been described in TM and SC cells and tissues. Integrin α5β1, one of the major fibronectin receptors, regulates fibronectin fibrillogenesis and TM contractility, while age-related loss of α5β1 promotes enhanced αvβ3 signaling and profibrotic response in TM cells (Johns et al., 2026; Johns et al., 2025). Integrin αvβ5, along with FAK signaling, mediates phagocytosis of TM cells, an activity negatively regulated by αvβ3-dependent crosstalk (Gagen et al., 2013). Vitronectin (VTN) mediated activation of integrin αvβ3 promotes SC formation while genetic inactivation of this signaling deteriorates SC morphogenesis leading to the development of ocular hypertension (Gu et al., 2024). These findings indicate that distinct integrin heterodimers coordinately regulate ECM remodeling, contractility, and phagocytosis in the TM and SC, providing a broader framework for investigating specialized functions of integrins in these tissues.

This review will focus on the integrin α4 and α9 subunits which are unique; they share roughly 39% sequence identity and represent a subfamily that does not depend on the classic RGD (Arg-Gly-Asp) motif but rather recognizes different, often acidic, peptide motifs (Xu et al., 2021; Young et al., 2001; Palmer et al., 1993). This subfamily is one of the newest and most specialized integrin families, based on evolutionary relationships, and only found in vertebrates (Hynes, 2002; Yokosaki et al., 1998). While traditionally studied in the context of leukocyte trafficking and wound healing, their expression in the TM and SC suggests a critical role in sensing the mechanical environment of the eye (Yan et al., 2020; Zhou et al., 1996; Holden et al., 2026; Balasubrama et al., 2024). Here we discuss the roles of α4β1 and α9β1 in regulating key TM and SC functions in outflow homeostasis.

## 2 Structure and function of integrin α4 and α9

Integrins α4 and α9 form a distinct subfamily by pairing with the β1 subunit resulting in α4β1 and α9β1 subunits (Xu et al., 2021).

Integrin α4β1, also known as very late antigen-4 (VLA-4), is primarily expressed in leukocytes (lymphocytes, eosinophils, monocytes, macrophages, natural killer cells, basophils, and mast cells), as well as in the developing vasculature and nervous system, tumor cells, and sites of wound healing (Hemler et al., 1987; Chan et al., 2001; Baiula et al., 2011; Mitroulis et al., 2015). Integrin α4β1 functions to mediate homing, trafficking, differentiation, activation, and cell survival (Hemler et al., 1987; Chan et al., 2001; Baiula et al., 2011; Mitroulis et al., 2015). The primary physiological ligands for α4β1 integrin include vascular cell adhesion molecule-1(VCAM-1) (Chan et al., 1992; Osborn et al., 1994), EDA domain of fibronectin (FN+EDA) (Liao et al., 2002; Shinde et al., 2015), osteopontin (OPN) (Bayless and Davis, 2001) and junctional adhesion molecule-B (JAM-B) (Cunningham et al., 2002; Ludwig et al., 2009). In ocular tissues, this integrin is responsible for maintaining the adhesion of TM cells and participating in crosstalk with other integrins to modulate the actin cytoskeleton (Filla et al., 2006; Faralli et al., 2011).

Structurally, the α9 subunit consists of a large N-terminal extracellular domain (which serves as the primary site for ligand binding), a transmembrane segment, and a short C-terminal cytoplasmic tail (Nawaz et al., 2015; Springer, 1997). Integrin α9β1 is widely expressed across the airway epithelium, keratinocytes, smooth, skeletal, and cardiac muscle cells, hepatocytes, neutrophils, osteoclasts and oocytes (Palmer et al., 1993; Singh et al., 2004; Rao et al., 2006; Staniszewska et al., 2007; Vjugina et al., 2009). It interacts with a diverse range of ECM ligands, such as the disintegrin and metalloprotease (ADAM) family, elastic microfibril interface-located protein1 (EMILIN1), vascular endothelial growth factor (VEGF), FN+EDA, tenascin-C (TNC), OPN, VCAM-1 and C-motif-ligand-1 (XCL1)/lymphotactin (Eto et al., 2002; Danussi et al., 2011; Vlahakis et al., 2005; Murtomaki et al., 2014; Saika et al., 2013). The interaction between these proteins and integrin α9β1 is fundamental to both cellular physiological activities and organismal growth and development.

**FIGURE 1:**
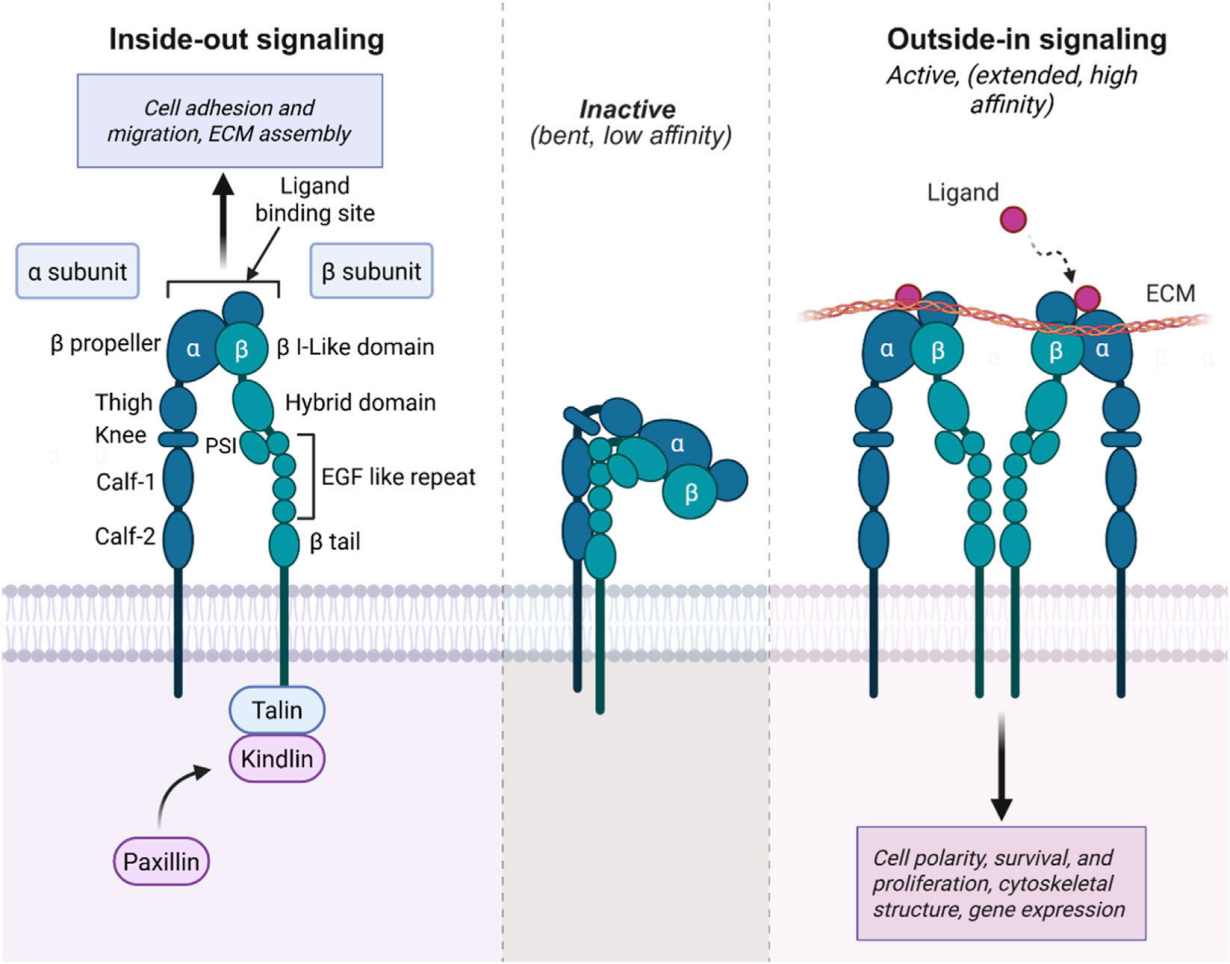
Schematic diagram of integrin heterodimers. Integrins are heterodimeric transmembrane receptors consisting of one α-subunit and one β-subunit. In an inactive state, the extracellular domains remain in a bent conformation with their cytoplasmic tails bound together. Upon activation, the integrin subunits undergo a conformational change, assume an upright conformation with their cytoplasmic tails separated. Integrin signaling can either be activated by extracellular ligands, which is called outside-in signaling, or can be activated by intracellular signals which changes the integrin into a high affinity conformation to facilitate ligand binding outside the cell, which is called inside-out signaling. Image created with Biorender.com.

A unique feature of integrins is that their activity involves very specific conformational changes in their α and β subunits (Li et al., 2017). In their unoccupied state, the extracellular domains remain in a bent conformation with their cytoplasmic tails bound together by a salt bridge. Upon activation, the integrin subunits undergo a conformational change and assume an upright conformation with their cytoplasmic tails separated (Figure 1). The integrin can either be activated by engaging its ligand which is called outside-in signaling or can be activated intracellularly by a secondary signaling pathway allowing the integrin binding to its ligand outside the cell which is called inside-out signaling.

## 3 Functions in trabecular meshwork and Schlemm’s canal

**FIGURE 2:**
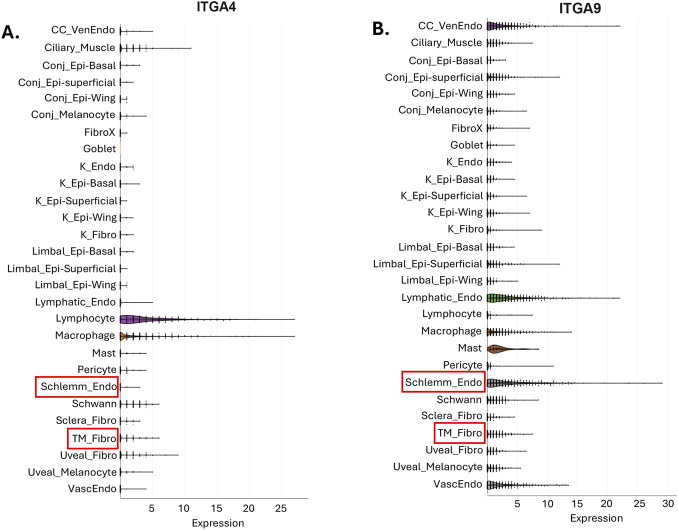
Tissue specific distribution of *ITGA4* and *ITGA9* expression in the human ocular anterior segment. Data derived from Single Cell Portal database (van Zyl et al., 2022). Violin plots of **(A)** *ITGA4* and **(B)** *ITGA9* relative expression in anterior segment structures. Expression in Schlemm’s canal (SC) endothelium and trabecular meshwork (TM) cells outlined in red boxes.

The TM and SC are highly mechanosensitive. The TM acts as a physical filter, while the inner wall of the SC forms giant vacuoles and pores to allow AH passage (Dautriche et al., 2015; Lewczuk et al., 2022). Integrin α4β1 has been reported to be expressed in human TM cells (HTM) (Yan et al., 2020; Zhou et al., 1996; Holden et al., 2026; Faralli et al., 2011; Zh et al., 1999; Schwinn et al., 2010; van Zyl et al., 2022) and mouse SC cells (Balasubrama et al., 2024), while integrin α9β1 has been shown to be expressed in mouse (Balasubrama et al., 2024; Park et al., 2014) and human (van Zyl et al., 2022) SC cells, and both integrins have also been found in human optic nerve head astrocytes (Morrison, 2006). Importantly, scRNA-Seq data of the human anterior chamber show tissue specific distribution of both integrin α4 (*ITGA4*) and α9 (*ITGA9*) in human TM and SC tissues (data derived from the publicly available Single Cell Portal platform (Figure 2) (van Zyl et al., 2022). These integrins function as “bidirectional transducers.” Through “inside-out” signaling they modulate the affinity for ligands, and “outside-in” signaling converts mechanical stretches, such as from changes in IOP, into biochemical signals that regulate cell contractility.

### 3.1 Mechanotransduction and cell contractility in TM

**FIGURE 3:**
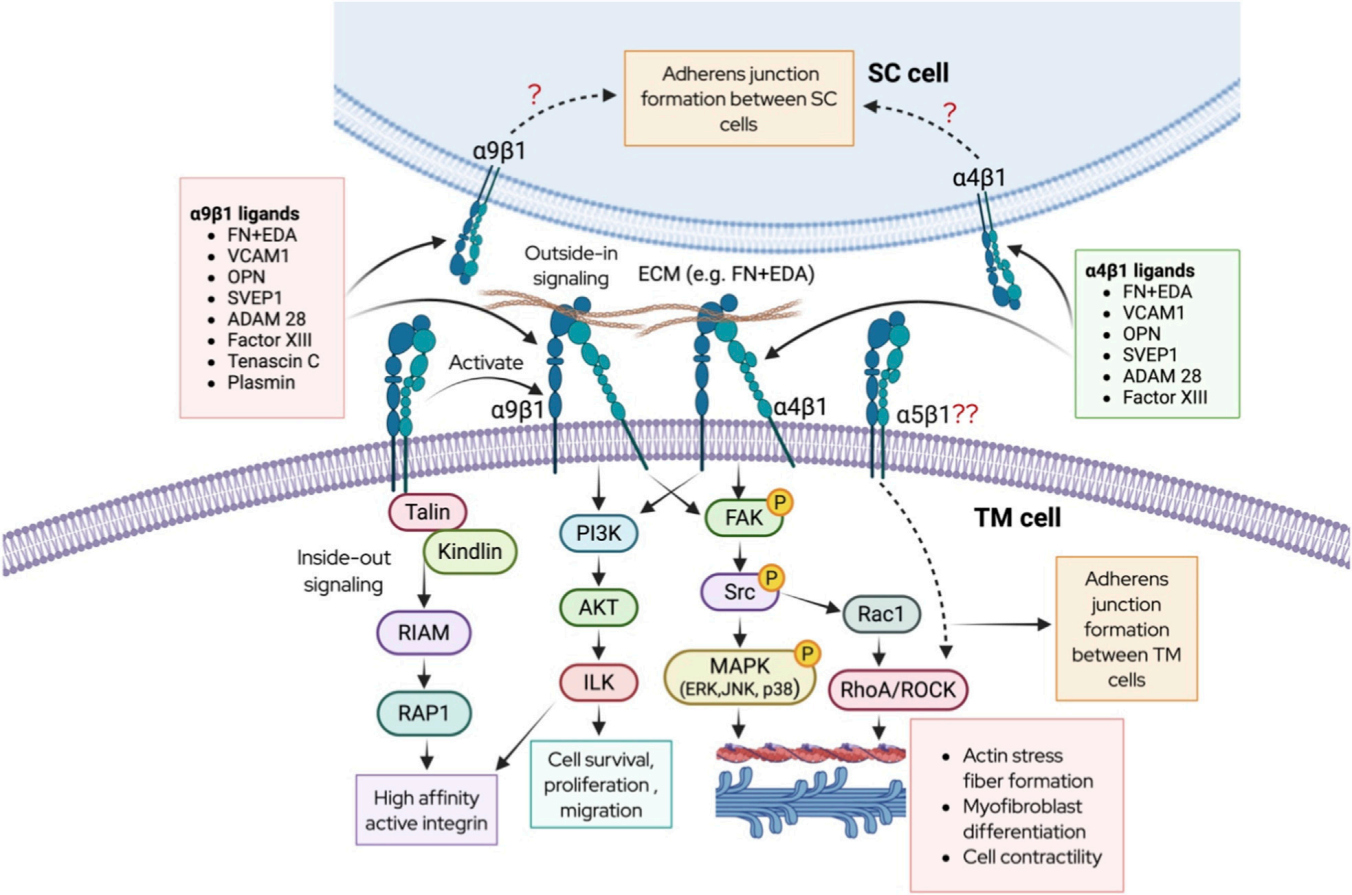
Proposed role of integrin α4β1 and α9β1 signaling in the TM and SC. Activation of integrin α4β1 and α9β1 receptors induces a cascade of events within cells. Talin along with kindlin bind to the β subunit, inducing a conformational change, which triggers the structural change of α subunit, allowing the integrin to bind to its ligand and initiate inside-out signaling. Extracellular ligands that bind to integrins initiate outside in signaling causing cytoskeletal changes via focal adhesion molecules including focal adhesion kinase (FAK), leading to cell proliferation, survival, differentiation, and migration. Integrins, along with other signaling molecules, like MAPK or RhoA/ROCK, can also regulate stress fiber formation, myofibroblast differentiation and cell contractility. Crosstalk between integrin α4β1 and/or α9β1 and other integrins like α5β1 may also enhance Rho activity and stabilizes adherens junctions (AJs) between TM cells as well as between SC cells. Image created with Biorender.com.

Integrin α4β1 and α9β1 are expressed in TM cells and α4β1 signaling has been shown to participate in regulating focal adhesion and actin stress fiber assembly (Peterson et al., 2005). In the healthy eye, the TM is relatively compliant, allowing it to distend under pressure. This distension is sensed by integrins, which signal the cell to adjust its contractility to maintain a constant IOP. Cooperative signaling between α4β1 and other integrins, such as α2β1 or α5β1, has been shown to modulate cell contractility (Faralli et al., 2019). Crosstalk between Heparin II (HepII)-bound α4β1 integrin and collagen-bound integrins (α1β1 or α2β1) has been reported to disrupt RhoA activity which is needed to stabilize adherens junctions (AJs) and stress fibers. In contrast, crosstalk between HepII-bound α4β1 integrin and α5β1 integrin fibronectin receptor enhance RhoA activity (Schwinn et al., 2010; Peterson et al., 2005). These observations signify the differential role of α4β1 integrin in regulating the contractile properties of TM cells which is associated with increased IOP and the development of POAG. Activation of the α4β1 integrin via the PRARI site in the HepII binding domain of FN also mediates the depolymerization of actin filaments and the disruption of adherens junctions in TM cells (Gonzalez et al., 2006; Gonzalez et al., 2009; Santas et al., 2003). Activation of α4β1 by the HepII domain of FN can also trigger crosstalk that enhances stress fiber formation and cell spreading in sub confluent TM cell cultures (Faralli et al., 2009; Peterson et al., 2005). Since integrin α9β1 has similar binding affinity for Hep II domain, it may also contribute to similar physiological changes in TM. In addition, it is likely that these signaling pathways effect the cell-cell communication between TM and SC cells (Figure 3).

### 3.2 The role of integrins in TM-SC crosstalk

SC is a unique “hybrid” endothelium with aspects of both blood and lymphatic vascular identity (Kizhatil et al., 2014; Balasubrama et al., 2024), and some of the lymphatic signaling molecules such as PROX1 and FLT4 have been identified to play a role in SC development and regulation (Aspelund et al., 2014; Park et al., 2014). A loss-of-function variant in *SVEP1*, which encodes an ECM protein necessary for lymphatic development and valve formation, was recently identified as a modifier of primary congenital glaucoma (PCG) disease (Young et al., 2020). Moreover, a common variant of *SVEP1* (rs61751937) has been identified as a risk allele for POAG via GWAS, suggesting a crucial role of *SVEP1* in IOP homeostasis (Gharahkhani et al., 2021). *SVEP1* serves as a ligand for integrin α9β1 and is known to be essential for SC development and maintenance (Morooka et al., 2017; Sato-Nishiuchi et al., 2012; Thomson et al., 2021). *SVEP1* expressed by neural crest-derived cells of the TM is essential for the development of the SC, which highly expresses integrin α9β1 (Thomson et al., 2021). As integrin α9β1 is also expressed in the TM (van Zyl et al., 2022), these data suggest that SVEP1-integrin α9β1 signaling may play a role in TM-SC crosstalk.

SC endothelial cells also express vascular endothelial cadherin (VE-cadherin), a classical cadherin found in adherens junctions that mediates homotypic cell–cell adhesion in vascular endothelia (Heimark et al., 2002). Crosstalk between integrins and cadherins can affect gene expression, organization of the cytoskeleton, as well as expression and localization of proteins in adherens junctions and focal adhesions, which together, can directly influence the permeability of the SC endothelial layer. For example, β1 integrin is required for the localization of VE-cadherin to intercellular junctions, as well as trafficking of VE-cadherin, and preservation of junctional integrity in retinal vasculature of mice (Yamamoto et al., 2015). Activation of integrin α9β1 is important in the formation of lymphatic-endothelial cell-cell and cell-matrix contacts during the development of SC in conjugation with SVEP1/Tie2 signaling complexes (Thomson et al., 2021). Activation of α5β1 integrin by FN has been shown to tighten lymphatic endothelial cell junctions (Henderson et al., 2021). In contrast, activation of αvβ3 integrin disrupts VE-cadherin cell junctions enhancing permeability in blood endothelial cells (Alghisi et al., 2009). Activation of α4β1 integrin via PRARRI sequence in FN has also been shown to disrupt adherens junctions in the TM, disrupt attachment of SC to the underlying basal membrane (BM), and increase AH outflow (Gonzalez et al., 2006). Increased availability of ECM proteins like FN+EDA in pathological condition like ocular hypertension may enhance α4β1 or α9β1 integrin engagement, modulating these cell-matrix and cell-cell interactions (Figure 3). Furthermore, α4β1 integrin has been reported to control the shear force-dependent contractile properties of endothelial cells (Goldfinger et al., 2008) which suggests that changes in integrin-mediated adhesion to the ECM likely influence endothelial contractility and cell–cell cadherin contacts. These data suggest that integrins and the ECM binding sites in the environment may have the capability to regulate adherens junction formation and regulate junctional rigidity and permeability in SC.

## 4 Functions in aqueous humor outflow and IOP regulation

The primary site of outflow resistance is the juxtacanalicular tissue (JCT), the inner most part of TM, and the inner wall of the SC (Dautriche et al., 2015). Integrin activity directly impacts the efficiency of AH drainage, known as outflow facility, in the eye (Filla et al., 2022). A decrease in outflow facility leads to elevated IOP, the primary risk factor for glaucoma.

Elevated IOP is often correlated with an activated conformation of integrins (Faralli et al., 2019). Integrins in the TM and SC act as functional modulators of aqueous humor outflow by regulating cytoskeletal organization and cellular mechanics (Filla et al., 2022). Previous studies show that activation of specific integrins, especially αvβ3, alters actin–myosin cytoskeletal dynamics and focal adhesion signaling, which in turn influences tissue stiffness and the mechanical behavior of the cells residing the outflow pathway tissues. More specifically, activation of αvβ3 integrin in perfused porcine anterior segment organ culture has been shown to decrease outflow facility and increase intraocular pressure (IOP), while genetic deletion of the β3 integrin subunit in mice lowers IOP, demonstrating that integrin activity can directly modulate outflow resistance (Faralli et al., 2019). While much focus has been placed on αvβ3, the α4 and α9 subfamilies are critical because they are expressed in the outflow pathway tissues and also respond to different ECM proteins, such as tenascin-C and FN+EDA, which are upregulated in both mechanically stretched and glaucomatous TM (Medina-Ortiz et al., 2013; Keller et al., 2013). Although changes in actomyosin contractility and cell mechanics are known to affect giant vacuole and pore formation at the SC inner wall (Swain et al., 2022), whether there is any direct connection between SC inner wall integrins like integrin α9 and pore density still needs to be investigated. Nonetheless, integrin-mediated modulation of the actin cytoskeleton provides a likely mechanism for integrins α4 and α9 to serve as dynamic physiological regulators of outflow facility and IOP.

## 5 Integrins and TLR4-TGFβ signaling crosstalk in the TM

**FIGURE 4:**
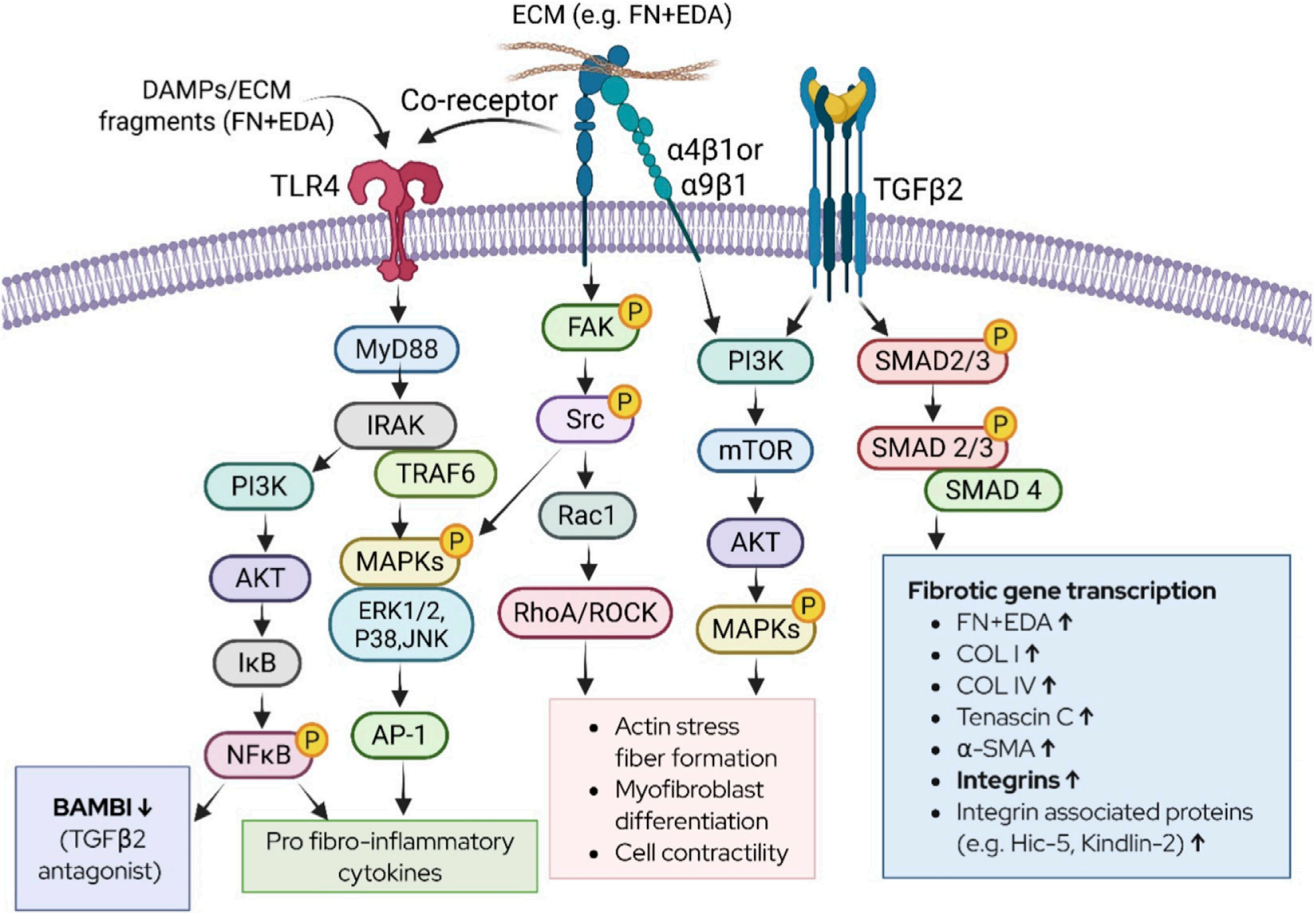
Proposed interaction of integrin α4β1 and α9β1 with TGFβ2-TLR4 signaling crosstalk. α4β1 and/or α9β1 serve as co-receptors for TLR4 and cooperate to recognize extracellular matrix changes and initiate a fibro-inflammatory cascade. TLR4 signaling also negatively regulates an endogenous TGFβ2 antagonist, BAMBI, which enhances uninhibited TGFβ2 signaling in the TM. TGFβ2 is known to induce expression of FN+EDA, a known ligand of TLR4 signaling. Together with either integrin α4β1 and/or α9β1 acting as a co-receptor, FN+EDA activation of TLR4 can create a feed forward loop of pro-fibrotic signaling. FN+EDA also promotes cooperative signaling between α4β1 and other integrins and activates RhoA-mediated stress fiber formation. TGFβ2 affects integrin-mediated cell adhesion and migration by regulating the expression of integrins, their ligands and integrin-associated proteins. Integrins, along with TGFβ2 signaling, regulate actin stress fiber formation, myofibroblast differentiation, and cell contractility. In summary, integrin α4β1 and α9β1 may play a crucial role in regulating pro-fibrotic signaling in the TM. Image created with Biorender.com.

TGFβ2 is the predominant isoform in the AH and is significantly elevated in POAG patients (Kraik et al., 2024) and is widely recognized as a profibrotic driving force involved in inducing TM dysfunction (Russell and Johnson, 2012; Vranka et al., 2018). In HTM cells, TGFβ2 robustly induces total FN and FN+EDA mRNA and protein expression as well as FN+EDA fibril formation. FN+EDA is also significantly elevated in glaucomatous TM tissues compared to normal controls, consistent with chronic TGFβ2 exposure in disease (Medina-Ortiz et al., 2013). Importantly, FN+EDA serves as a ligand for both integrin α4β1 and α9β1 (28). Myofibroblast differentiation by TGFβ1 is dependent on cell adhesion and integrin signaling (Thannickal et al., 2003). TGFβ1 upregulates expression of integrins α4, α5 and β1, an effect that is associated with autophosphorylation/activation of FAK signaling in lung fibroblasts (Thannickal et al., 2003). Ligand engagement of integrins α4β1 and α9β1 activates focal adhesion-associated signaling pathways, including FAK/Src, paxillin-dependent Rac/Rho-family GTPases, MAPK, and in a cell-type-dependent manner PI3K/Akt (Young et al., 2001; Yokosaki et al., 1998; Liao et al., 2002; Shinde et al., 2015; Liu et al., 2002). These signaling pathways are likely to intersect with downstream TGFβ signaling and thereby promote extracellular matrix remodeling, myofibroblast differentiation, and fibrosis (Figure 4).

Toll-like receptor 4 (TLR4), a member of the Toll-like receptor family, has emerged as another important mediator of fibrotic signaling. TLR4 was originally identified as the receptor for lipopolysaccharide (LPS) (Poltorak et al., 1998; Hoshino et al., 1999). In addition to pathogen-associated ligands, TLR4 can be activated by endogenous damage-associated molecular patterns (DAMPs) generated during tissue injury, cell stress, ECM remodeling, and oxidative stress (Piccinini and Midwood, 2010). Increasing evidence links DAMP-mediated TLR4 activation to fibrosis and to the regulation and production of ECM proteins. Several DAMPs, including FN+EDA, low-molecular-weight hyaluronan, and tenascin-C, have been shown to activate TLR4 and enhance TGFβ signaling, thereby amplifying downstream profibrotic responses.

Among these DAMPs, FN+EDA has been shown to induce fibrotic phenotypes in TM leading to ocular hypertension. Constitutive expression of FN+EDA in mouse models amplifies TGFβ2-induced ocular hypertension, and this effect is blocked in *Tlr4* mutant mice, indicating that FN+EDA-TLR4 signaling is required for TGFβ2-induced TM damage and elevated IOP (Hernandez et al., 2017). Moreover, TLR4 signaling negatively regulates an endogenous TGFβ2 antagonist, BAMBI, which enhances uninhibited TGFβ2 signaling in the TM (Hernandez et al., 2018). Therefore, TGFβ2 induced FN+EDA expression establishes a feed forward signaling loop in which FN+EDA activates TLR4, leading to sustained ECM production and progressive fibrotic response. While inhibition of TLR4 signaling reduces TGFβ2-induced ECM deposition in TM cells (Figure 4) (Hernandez et al., 2017; Mzyk et al., 2022).

Emerging evidence further suggests that integrins α4β1 and α9β1 may cooperate with TLR4 signaling in response to FN+EDA. Recent study in fibroblasts shows that, integrin α4β1 functions as a TLR4 coreceptor initiating EDA-dependent pro-fibrotic responses (Kelsh-Lasher et al., 2017). In addition, FN+EDA also promotes cooperative signaling between α4β1 and other integrins and activates RhoA-mediated stress fiber formation in HTM cells (Peterson et al., 2005). These findings suggest that FN+EDA may simultaneously engage TLR4 and integrins α4β1 and/or α9β1, with these integrins acting as co-receptors, to amplify profibrotic signaling within the TM. However, the specific role of α4β1 and α9β1 integrins in TGFβ2-TLR4 mediated pro-fibrotic response in the TM and SC is yet to be revealed.

## 6 Role of integrins in fibrotic response

Human dermal fibroblasts plated on FN+EDA shows fibrotic ECM and contractile phenotypes such as increased stress fibers, myosin-light chain phosphorylation, fibronectin synthesis, and fibrillogenesis, and importantly this effect was dependent on α4β1 engagement with FN+EDA (Shinde et al., 2015). In addition, interaction of FN+EDA with α4β1 cooperates with TLR4 to increase pro-fibrotic gene expression in fibroblasts, including further fibronectin production, and promotes a contractile phenotype with increased actin stress fibers and myosin activation (Kelsh-Lasher et al., 2017).

Increased matrix stiffness is sensed through integrin-mediated mechanotransduction, leading to enhanced α-smooth muscle actin (α-SMA) expression and myofibroblast differentiation. This has been demonstrated in fibroblasts, where FN+EDA promotes a pro-fibrotic phenotype characterized by increased α-SMA expression, enhanced collagen deposition, elevated contractility, and activation of focal adhesion kinase (FAK) signaling and these effects depend on the binding of the EDA domain to specific integrins such as α4β1 (Kohan et al., 2010). Consistently, FN+EDA has been reported to interact with either α4β1, or α9β1 to trigger early stages of myofibroblast formation, leading to increased α-SMA expression and contractility in fibroblast systems (Kohan et al., 2010; Faralli et al., 2023). Other mechanobiology studies show that substrate stiffness enhances integrin-linked FAK/ROCK signaling and α-SMA incorporation into stress fibers (Hinz et al., 2001; Wipff et al., 2007).

Integrin α9β1 has also been reported to bind with FN+EDA via the EDGIHEL sequence and is required for proper fibronectin matrix assembly in primary human lymphatic endothelial cells during valve morphogenesis. Cells lacking α9 have disorganized FN fibrillar matrices, demonstrating that α9β1 directly influences matrix assembly (Bazigou et al., 2009). Smooth muscle cells lacking α9 show reduced β-catenin activity and lower transcription of MMP-2 and MMP-9, indicating that α9β1 engagement can regulate Matrix Metalloproteinase (MMP) expression pathways (Jain et al., 2021). MMP regulation is critical in matrix remodeling and fibrosis, and this result supports the role of α9β1 integrin in modulating ECM turnover signals.

In human TM cells, TGFβ2 robustly increases total FN and FN+EDA matrix deposition and FN+EDA is upregulated in the glaucomatous HTM tissues (Medina-Ortiz et al., 2013). Importantly, we have shown constitutively active FN+EDA induces elevated IOP in mice and FN+EDA increases ECM production in TM cells (Hernandez et al., 2017; Mavlyutov et al., 2022a; Mavlyutov et al., 2022b; Roberts et al., 2020), providing a stiffened ECM environment that can facilitate integrin mechanosignaling and contribute to contractile phenotypes consistent with fibrotic responses mediated by integrins α4 and α9 in other tissues.

## 7 Ligands of integrin α4β1 and α9β1 relevant to glaucoma pathogenesis

To elucidate how α4β1 and α9β1 regulate TM homeostasis and fibrosis, it is essential to understand their ligand profiles (Table 1). These integrins share structural similarities as well as many ligands, although there are distinct affinities for some ECM components. Both α4β1 and α9β1 share affinity to ligands including FN+EDA (Xu et al., 2021; Liao et al., 2002; Shinde et al., 2015; Kohan et al., 2010), VCAM-1 (Taooka et al., 1999; Takahashi et al., 2000), OPN (Nishimichi et al., 2009; Yokosaki et al., 1994), SVEP-1 (Morris et al., 2022), ADAM-28 (Bri et al., 2003; Bridges et al., 2005; Bridges et al., 2002), Factor XIII (Takahashi et al., 2000) which function in processes such as ECM remodeling, cell adhesion, and/or cell-contractility upon integrin activation in TM and SC cells. However, integrin α9β1 can also bind ligands including tenascin-C (Yokosaki et al., 1998; Taooka et al., 1999; Yokosaki et al., 1994) and plasmin (Majumdar et al., 2004) which are associated with ECM remodeling. These data suggest there is likely some overlapping function, but also distinct roles of α4β1 and α9β1 in TM and SC homeostasis and pathology.

**TABLE 1:** Ligands of integrin α4β1 and α9β1 relevant to glaucoma pathogenesis.

Ligand	α4β1	α9β1	Functions/Mechanistic roles (glaucoma relevance)	Experimental Reference(s)
FN+EDA	+	+	Promotes myofibroblast-like phenotype, stress fiber formation, ECM assembly; implied mechano-signaling that could support ECM deposition, stiffness responses	Xu et al. (2021), Liao et al. (2002), Shinde et al. (2015), Kohan et al. (2010)
VCAM-1	+	+	Mediates cell–cell adhesion; may influence cell tension and junctional integrity under mechanical stress	Taooka et al. (1999), Takahashi et al. (2000)
Osteopontin (OPN)	+	+	Promotes adhesion/migration; possible matrix remodeling involvement that could be relevant to fibrotic change	Nishimichi et al. (2009), Yokosaki et al. (1994)
Tenascin-C	–	+	Alters adhesion dynamics; implicated in matrix remodeling, cell migration, potential influence on TM mechanobiology	Yokosaki et al. (1998), Taooka et al. (1999), Yokosaki et al. (1994)
SVEP1	+	+	Modulates contractility via calcium signaling and RhoA, suggests a role in cell contractile responses affecting tissue tension	Morris et al. (2022)
ADAM28	+	+	Cell adhesion; potential influence on cell–matrix interaction and mechano-sensing	Bri et al. (2003), Bridges et al. (2005), Bridges et al. (2002)
Plasmin	–	+	Potential roles in matrix proteolysis and remodeling	Majumdar et al. (2004)
Factor XIII	+	+	These ECM crosslinkers influence matrix stability, mechanistically relevant to stiffness	Takahashi et al. (2000)

## 8 Therapeutic strategies targeting integrin α4 and α9

Given the role of integrin α4β1 and α9β1 in driving a fibrotic cascade and increasing outflow resistance in the TM and SC, they have emerged as potential therapeutic targets. Unlike broad-spectrum TGFβ signaling pathway inhibitors, which can have significant systemic side effects, targeting specific integrins might allow for more localized and pathway-specific modulation of the TM environment.

### 8.1 Small-molecule inhibitors and peptides

Research into integrin inhibition has yielded several candidates that could potentially be repurposed for ocular hypertension treatment.

Integrin α4β1 antagonists (e.g., Natalizumab and small molecules): While Natalizumab is a systemic monoclonal antibody of integrin α4β1 used in multiple sclerosis, small-molecule inhibitors are also being explored to disrupt the binding between TM cells and the CS-1/HepII domains of FN. Engagement of the α4β1 integrin by specific ECM domains (e.g., HepII) has been shown to promote disassembly of actin stress fibers and reduce cell contractility in HTM cells, an effect associated with increased outflow facility, previously observed in monkey and human eye anterior segment perfusion models (Gonzalez et al., 2009; Santas et al., 2003).

Integrin α9β1 specific inhibitors: Integrin α9β1 has been proposed as a therapeutic target in several refractory diseases (Xu et al., 2021; Schreiber et al., 2009; Asano et al., 2014). Blocking integrin α9β1 with specific monoclonal antibodies (e.g., 55A2C) reduces pathological outcomes in animal models like collagen-induced arthritis (Xu et al., 2021). These antibodies interfere with α9β1 binding to its ECM ligands (such as FN+EDA) in vascular remodeling and autoimmune disease models. Recent investigation shows that α9β1 binds the FN+EDA sequence and other non-RGD ECM motifs and that blocking this interaction (with antibodies or peptides) alters downstream cell behavior. An anti-α9 antibody in vascular smooth muscle cells, reduced neointimal hyperplasia, potentially by inhibiting α9β1 binding to FN+EDA like sequences (Jain et al., 2021).

### 8.2 Disrupting the FN+EDA-integrin interaction

The FN+EDA drives fibrotic responses in multiple tissue types (e.g., lung, skin) largely by acting as a ECM component that engages integrin receptors and TLR4, promoting myofibroblast activation, extracellular matrix production, and sustained fibrosis (Serini et al., 1998). In human dermal fibroblast cultures, a blocking monoclonal antibody (IST-9) against FN+EDA, reduces incorporation of latent TGFβ binding protein-1 (LTBP-1) into the ECM, leading to lower activation of latent TGFβ1 which is a key mediator of fibrosis (Klingberg et al., 2018). This supports the idea that targeting FN+EDA can inhibit TGFβ-dependent fibrotic signaling in TM. A recent study shows that AF38Pep, a high-affinity polypeptide, binds with FN+EDA C-C′ loop and prevents its interaction with integrins (specifically α4β1/α4β7) in fibroblasts, which inhibits FN+EDA-mediated myofibroblast differentiation and profibrotic gene expression. The peptide was designed to have high-affinity for FN+EDA C-C′ loop binding cleft within integrins α4β1 and α4β7 (Zhang et al., 2021). This suggests that blocking FN+EDA receptor engagement with integrins can attenuate fibrotic fibroblast activation. Another study shows that systemic treatment with an anti-FN+EDA antibody, F8, reduced pathologic tissue remodeling and associated fibrosis in a pulmonary hypertension model, suggesting this antibody can functionally block FN+EDA associated processes *in vivo* (Singerer et al., 2024). All these studies suggest that disrupting FN+EDA and integrin interaction could be a potential therapeutic strategy to reduce fibro-inflammatory response in the TM.

## 9 Conclusion

Integrins α4 and α9 have emerged as important regulators of TM biology, functioning at the interface between ECM remodeling and intracellular signaling pathways that regulate cytoskeletal dynamics, contractility, and inflammatory responses. Evidence indicates that α4β1 participates in integrin-TLR4 crosstalk and modulates RhoA-dependent actin organization, thereby possibly influencing TM stiffness and outflow resistance. Meanwhile, α9β1 through its ability to bind non-RGD ligands such as FN+EDA, tenascin-C, and OPN, represents a possible mechanism of regulating ECM driven fibrotic and inflammatory responses. Therefore, integrin α4 and α9 occupy a strategic position in TM homeostasis by integrating mechanical, biochemical, and inflammatory cues from the ECM. A deeper understanding of their ligand specificity, signaling crosstalk, and temporal regulation in glaucomatous tissue will be essential for defining whether selective modulation of these pathways can restore normal outflow and provide novel, targeted therapies for glaucoma.
